# Enhancement of gut barrier integrity by a *Bacillus subtilis* secreted metabolite through the GADD45A‐Wnt/β‐catenin pathway

**DOI:** 10.1002/imt2.70005

**Published:** 2025-02-23

**Authors:** Shiqi Liu, Peiran Cai, Wenjing You, Mingshun Yang, Yuang Tu, Yanbing Zhou, Teresa G. Valencak, Yingping Xiao, Yizhen Wang, Tizhong Shan

**Affiliations:** ^1^ College of Animal Sciences Zhejiang University Hangzhou China; ^2^ Key Laboratory of Molecular Animal Nutrition (Zhejiang University) Ministry of Education Hangzhou China; ^3^ Zhejiang Key Laboratory of Nutrition and Breeding for High‐quality Animal Products Hangzhou China; ^4^ Austrian Agency for Health and Food Safety Vienna Austria; ^5^ State Key Laboratory for Managing Biotic and Chemical Threats to the Quality and Safety of Agro‐products, Institute of Agro‐product Safety and Nutrition Zhejiang Academy of Agricultural Sciences Hangzhou China

**Keywords:** 2‐hydroxy‐4‐methylpentanoic acid, GADD45A, intestinal barrier, metabolites, probiotics, Wnt/*β*‐catenin signaling

## Abstract

Inflammatory bowel disease (IBD) represents a significant challenge to global health, characterized by intestinal inflammation, impaired barrier function, and dysbiosis, with limited therapeutic options. In this study, we isolated a novel strain of *Bacillus subtilis* (*B. subtilis*) and observed promising effects in protecting against disruption of the gut barrier. Our findings indicate that the enhancement of intestinal barrier function is primarily attributed to its metabolites. We identified a novel metabolite, 2‐hydroxy‐4‐methylpentanoic acid (HMP), derived from *B. subtilis*, that significantly improved intestinal barrier function. We also show that growth arrest and DNA damage 45A (GADD45A) is a key regulator of mucosal barrier integrity, which is activated by HMP and subsequently activates the downstream Wnt/β‐catenin pathway. Our findings potentially contribute to the development of probiotics‐derived metabolites or targeted “postbiotics” as novel therapeutics for the treatment or prevention of IBD and other diseases associated with intestinal barrier dysfunction.

## Highlights

- *Bacillus subtilis* (*B. subtilis*) protects against lipopolysaccharide‐induced acute intestinal injury and inflammation.
- *B. subtilis* alleviates gut microbiota imbalance and increases the relative abundance of *Akkermansia*.
- 2‐hydroxy‐4‐methylpentanoic acid (HMP) produced by *B. subtilis* enhances intestinal epithelial barrier integrity.
- Growth arrest and DNA damage 45A (GADD45A) is a novel regulator of the intestinal epithelial barrier.
- HMP improves intestinal epithelial barrier integrity via the GADD45A‐Wnt/β‐catenin pathway

## Graphical abstract

*Bacillus subtilis* (*B. subtilis*) and its metabolite 2‐hydroxy‐4‐methylpentanoic acid alleviated lipopolysaccharide (LPS)‐induced intestinal epithelial barrier damage via the growth arrest and DNA damage 45A (GADD45A)‐Wnt/β‐catenin axis. LPS treatment led to a significant disruption of gut homeostasis. *B. subtilis* administration could restore gut homeostasis by alleviating inflammatory responses, increasing the abundance of beneficial bacteria, and enhancing the intestinal epithelial barrier.

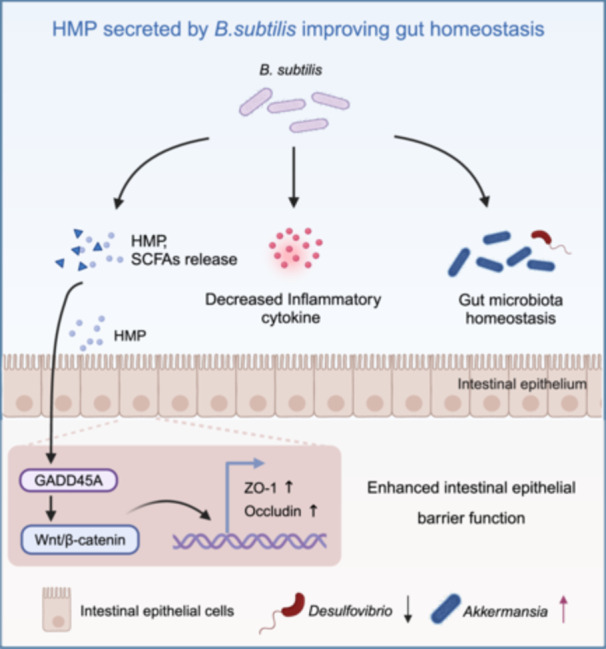

## INTRODUCTION

Intestinal homeostasis depends on complex interactions among gut microbiota, the intestinal epithelium, and the host immune system [1, 2]. Any perturbation of this equilibrium can precipitate a cascade of intestinal pathologies, with inflammatory bowel disease (IBD) being one of the most prevalent and refractory conditions. IBD, encompassing both Crohn's disease (CD) and ulcerative colitis (UC), is characterized by chronic and episodic inflammation within the gastrointestinal (GI) tract. The hallmarks of IBD include immune dysregulation, abnormal cytokine secretion, damage to the intestinal barrier, and imbalance of intestinal microbiota [1, 3]. The intestinal barrier is an interface between gut microbiota and the mucosal immune system, contributing to maintaining mucosal homeostasis [3, 4]. Dysregulation of the intestinal barrier function can lead to a condition known as “leaky gut” or increased intestinal permeability [5]. The intestinal epithelium, a monolayer of epithelial cells, is interconnected through tight junctions to form a physical barrier that efficiently precludes the translocation of noxious agents, such as pathogens and endotoxins, across the intestinal mucosa into the bloodstream [5, 6, 7]. These adjacent epithelial cells are linked to proteins that form tight junctions, including occludin, zonula occludens (ZOs), and junctional adhesion molecules, to preserve the integrity of the intestinal barrier [8]. The integrity of the intestinal barrier is a fundamental prerequisite for maintaining intestinal homeostasis [9].

Probiotics that regulate intestinal barrier function can maintain intestinal homeostasis while effectively treating intestinal diseases [10]. Numerous studies have shown that oral administration of probiotics can protect the integrity of the intestinal barrier and can alleviate intestinal inflammation during colitis [11, 12, 13, 14, 15, 16]. As an example, oral gavage with live *Alistipes onderdonkii* mitigated dextran sulfate sodium (DSS)‐induced colitis in mice by activating the aromatic hydrocarbon receptor (AhR) signaling pathway [11]. A recent study demonstrated that *Latilactobacillus sakei* CCFM1267 significantly restored colon length and tight‐junction protein expression while ameliorating the disease process in DSS‐induced murine colitis [12]. Additionally, *Bacteroides ovatus* effectively improved intestinal barrier integrity, reduced systemic inflammation, and decreased insulin resistance in mice fed a high‐fat diet [17]. These studies provide valuable data for the therapeutic use of probiotics during IBD. *Bacillus subtilis (B. subtilis)*, a Gram‐positive bacterium known for forming spores, is recognized as a Generally Recognized As Safe (GRAS) species [18]. It has been extensively studied and utilized in producing various biochemicals [19]. Some strains within the *Bacillaceae* family have been proven to be safe for human ingestion. For example, five *B. subtilis* strains have attained the status of “generally regarded as safe” (GRAS) by the US‐based Food and Drug Administration, pointing to regulatory approval for their incorporation into food products [20]. The European Food Safety Authority has granted *B. subtilis* a status on the qualified presumption of safety list, allowing its utilization within the food industry [21]. *B. subtilis* indeed has great potential for the treatment of intestinal diseases. A previous study showed that *B. subtilis* M6 improved intestinal barrier and antioxidant capacity via altering gut microbiota in a broiler model [22]. *B. subtilis* also inhibited intestinal inflammation and oxidative stress by regulating gut microbiota in laying hens [23]. Additionally, *B. subtilis* has been clinically identified to alleviate gas‐related gastrointestinal symptoms in participants with functional dyspepsia [24], as well as in healthy participants [20, 25, 26]. However, it remains unknown whether *B. subtilis* affects acute intestinal injury and mucosal barrier dysfunction. Although some *B. subtilis* strains have been observed to be safe and effective while improving intestinal barrier function, the underlying molecular mechanisms are largely unknown.

Here, we aimed to evaluate the role of a novel strain of *B. subtilis* in mitigating the disruption of the gut barrier and to elucidate the underlying molecular mechanisms. In this study, we isolated a novel strain of *B. subtilis* and identified that, in addition to its anti‐inflammatory activities, it maintains homeostasis of gut microbiota in a LPS‐induced intestinal injury model. Moreover, *B. subtilis* significantly alleviates dysfunction of the intestinal barrier. Our findings suggest that the enhancement of intestinal barrier function occurs primarily via its metabolites. We identified a novel metabolite, 2‐hydroxy‐4‐methylpentanoic acid (HMP), secreted by *B. subtilis*, which can significantly improve intestinal barrier integrity. Furthermore, HMP improved mucosal barrier integrity by activating the growth arrest and DNA damage 45A (GADD45A)‐Wnt/*β*‐catenin signaling pathway. Altogether, our study provides novel insights into the therapeutic potential of *B. subtilis* and its metabolites for IBD and other diseases associated with intestinal barrier dysfunction.

## RESULT

### *B. subtilis* protects against LPS‐induced acute intestinal injury and inflammation

**Figure 1:**
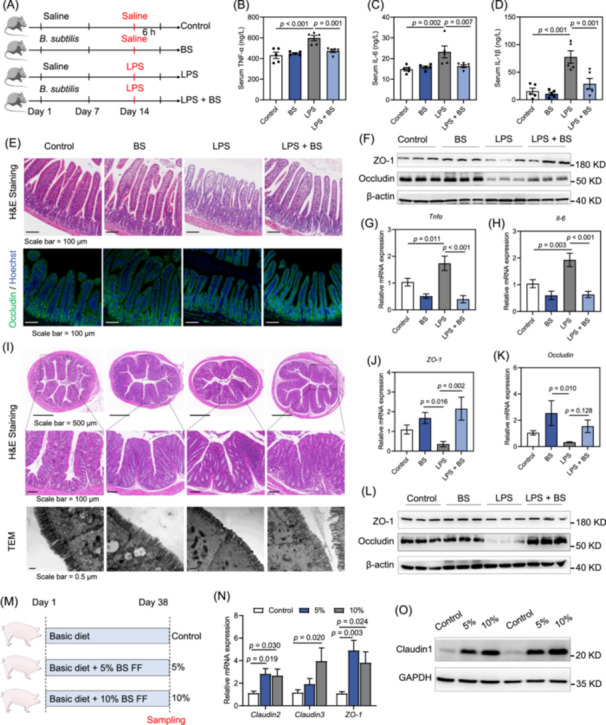
*B. subtilis* treatment alleviates LPS‐induced experimental intestinal epithelial barrier damage and inflammation in C57BL/6 mice. (A) Experimental design of the mouse model (mouse experiment 1). (B–D) Serum levels of inflammatory cytokines TNF‐α (B), IL‐6 (C), and IL‐1β (D) (*n* = 5). (E) Representative jejunal histology images by H&E staining and immunofluorescence of Occludin. (F) Western blot analysis of ZO‐1 and Occludin in jejunum. (G) Relative mRNA levels of *Tnfα* in jejunal tissues (*n* = 5). (H) Relative mRNA levels of *Il‐6* in jejunal tissues (*n* = 5). (I) Representative colon histology images by H&E staining and morphology of intestinal villi by TEM. (J) Relative mRNA levels of *ZO‐1* in colon tissues (*n* = 5). (K) Relative mRNA levels of *Occludin* in colon tissues (*n* = 5). (L) Western blot analysis of ZO‐1 and Occludin in colon tissues. (M) Experimental design of the pig model. (N) Relative mRNA levels of *Claudin2*, *Claudin3*, and *ZO‐1* in jejunal tissues (*n* = 6). (O) Western blot analysis of Claudin1 in colon tissues. Statistical analysis was performed using a one‐way analysis of variance (ANOVA) followed by Fisher's least significant difference test. The data are shown as means ± SEM. *B. subtilis*/BS, *Bacillus subtilis*; FF, Fermented feed; H&E, hematoxylin and eosin; IL‐6, interleukin‐6; IL‐1β, interleukin‐1β; LPS, lipopolysaccharide; mRNA, messenger RNA; SEM, standard error of measurement; TEM, transmission electron microscopy; TNF‐α, tumor necrosis factor α.

We investigated the possible role of *B. subtilis* during LPS‐induced acute intestinal injury. Following oral administration with sterile saline or *B. subtilis* for 14 days, 10‐week‐old male C57BL/6J mice were intraperitoneally injected with LPS (Figure 1A). There were no significant differences in body and spleen weights among the four groups (Figure S1A–C). LPS significantly increased the levels of serum tumor necrosis factor α (TNF‐α), interleukin‐6 (IL‐6), and interleukin‐1β (IL‐1β), while *B. subtilis* treatment significantly decreased serum TNF‐α, IL‐6, and IL‐1β (Figure 1B–D). Furthermore, LPS induced the atrophy of intestinal villi according to the immunofluorescence results, as well as a decrease of Occludin (Figure 1E). *B. subtilis* treatment restored the morphology of intestinal villi and Occludin (Figure 1E). Consistently, LPS treatment lowered the protein levels of ZO‐1 and Occludin, but *B. subtilis* significantly rescued the expression of ZO‐1 and Occludin, as shown by western blot (Figure 1F, Figure S1D). LPS significantly increased the mRNA expression of *Tnfα* and *Il‐6* in the jejunum, while mRNA expression of *Tnfα* and *Il‐6* was significantly lower in the LPS + BS group (Figure 1G,H). Similarly, LPS caused intestinal villi atrophy, damage to the intestinal barrier, and inflammatory cell infiltration in mouse colon tissues. *B. subtilis* treatment ameliorated the damage to the intestinal villi and inflammatory cell infiltration induced by LPS (Figure 1I). Compared to the LPS group, mRNA expression levels of *ZO‐1* and *Occludin* were increased by LPS + BS treatment (Figure 1J,K). Relating to protein levels, LPS + BS treatment restored the expression of Occludin in the LPS‐induced acute intestinal injury model (Figure 1L, Figure S1E). In addition, we observed that LPS + BS treatment significantly reduced mRNA expression of *Tnα* and *Il‐6* (Figure S1F).

To investigate the role of *B. subtilis* in pigs, we prepared fermented feed (FF) derived from *B. subtilis* and conducted experiments on pig tissues (Figure 1M). Although there were no significant differences in the morphology of the intestinal villi (Figure S1G), FF derived from *B. subtilis* (BS FF) significantly increased mRNA expression of *Claudin2*, *Claudin3*, and *ZO‐1* (Figure 1N). Relating to protein levels, BS FF significantly upregulated the protein expression of Claudin1 (Figure 1O). Additionally, supplementing 10% FMF significantly increased serum IgA levels compared to the control group (Figure S1H). Supplementing 10% BS FF significantly raised the activity of superoxide dismutase (SOD) in colon tissues (Figure S1I). Thus, *B. subtilis* likely plays a role in enhancing the integrity of the intestinal epithelial barrier and mitigating inflammatory processes.

### *B. subtilis* treatment alters gut microbial composition and increases the abundance of “beneficial” bacteria

Given the key role of gut microbiota for host immunity and intestinal homeostasis, we pretreated with *B. subtilis* and observed the effects on the gut microbiome using the 16S ribosomal ribonucleic acid (rRNA) sequencing technique. Principal coordinate analysis (PCoA) indicated significant differences in bacterial communities between the LPS group and the Ctrl group. LPS + BS and LPS treatment had a small effect on the bacterial communities (Figure S2A). In the colon, *Bacteroidota*, *Firmicutes*, *Verrucomicrobiota*, and *Proteobacteria* were the most abundant phyla, with an average abundance exceeding 90% (Figure S2B). In addition, the levels of *Verrucomicrobiota* in the LPS group were lower, while LPS + BS treatment rescued their abundance (Figure S2B). At the genus level, *Akkermansia* and *Stenotrophomonas* were more abundant (Figure S2C). Interestingly, the abundance of *Akkermansia*, *Parabacteroides*, *Alistipes*, and *Alloprevotella* in the LPS + BS group was significantly higher compared to the LPS group (Figure S2C).

To investigate phylogenetic relationships among species at the phylum and genus levels, representative sequences of the top 100 genera were obtained through multiple sequence alignment, and a phylogenetic tree was constructed (Figure S2D). We observed that the most abundant genus was *Firmicutes* (Figure S2D). Notably, the abundance of *Akkermansia*, an important probiotic for regulating intestinal barrier function [27], was decreased by LPS treatment, while LPS + BS treatment restored its abundance (Figure S2D). To elucidate the effects of *B. subtilis* on the composition and structural attributes of the colonic microbiota, a heat map was used to illustrate the relative abundance of 35 genera (Figure S2E). *Desulfovibrio*, *IIeibacterium*, and *Lachnoclostridium* were enriched in the LPS group, and *Bifidobacterium*, *Allobaculum*, *Alloprevotella*, and *Parabacteroides* were enriched in the LPS + BS group (Figure S2E). Consistently, LPS treatment decreased the abundance of *Akkermansia*, and LPS + BS treatment restored its abundance (Figure S2E). Additionally, LPS treatment increased the abundance of *Desulfovibrio*, while LPS + BS treatment decreased its abundance (Figure S2E). Based on the Kyoto Encyclopedia of Genes and Genomes (KEGG) database, we analyzed the microbial functional differences and predicted microbial metabolic functions with the Phylogenetic Investigation of Communities by Reconstruction of Unobserved States (PICRUSt). We had predicted that compared with the LPS group, the function of pyruvate metabolism, sphingolipid metabolism, phosphonate and phosphinate metabolism, selenocompound metabolism, d‐alanine metabolism, nitrogen metabolism, and vitamin B6 metabolism would be significantly stronger, while the function of tropane piperidine and pyridine alkaloid biosynthesis, fat digestion and absorption, and primary bile acid biosynthesis would be lower in LPS + BS group (Figure S2F). Likewise, compared to the LPS group, the function of citrate cycle and membrane trafficking was predicted to be significantly stronger in the LPS + BS group (Figure S2F).

To examine the associations between significantly different genera and gene expression levels of tight junction proteins and inflammatory factors, a correlation analysis was performed (Figure S2G). The relative abundance of *Akkermansia* showed strong positive correlations with the expression of *Occludin* and was negatively associated with the expression of *TNF‐α* and *Il‐6* (Figure S2G). The relative abundance of the *Limosilactobacillus* was positively correlated with the expression of *ZO‐1* while being negatively correlated with the expression of *TNF‐α* and *Il‐6* (Figure S2G). Additionally, some genera, including *Desulfovibrio*, *Oscillibacter*, and *Negativibacillus*, were strongly positively correlated with the expression of *TNF‐α* and *Il‐6* and negatively correlated with the expression of *ZO‐1* (Figure S2G). Collectively, we show that *B. subtilis* exposure improved the abundance of beneficial microbiota, reducing dysbiosis induced by LPS, which is closely related to homeostasis of the intestinal epithelial barrier.

### Protective effects of *B. subtilis* against LPS‐induced acute intestinal injury are primarily associated with metabolites

**Figure 2:**
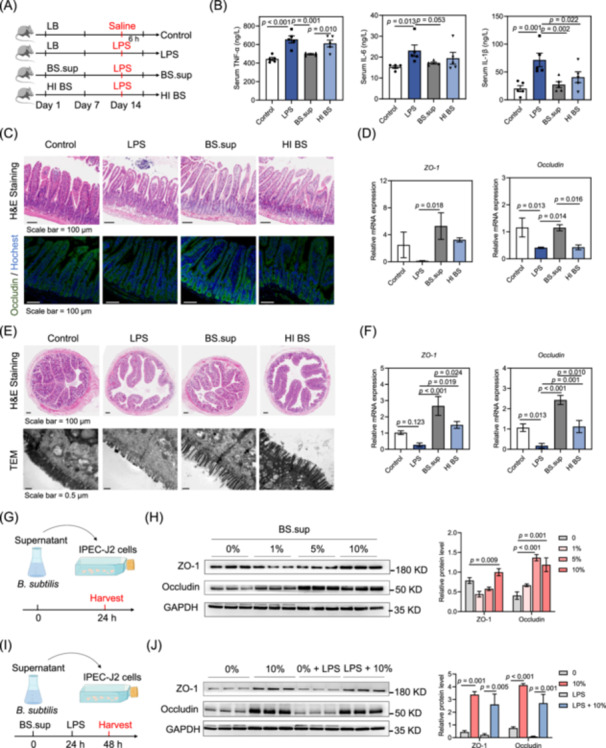
The protective effect of *B. subtilis* primarily relies on its metabolites. (A) Experimental design of the mouse model. (B) Serum levels of inflammatory cytokines TNF‐α, IL‐6, and IL‐1β (*n* = 5). (C) Representative jejunal histology images by H&E staining and immunofluorescence of Occludin. (D) Relative mRNA levels of *ZO‐1* and *Occludin* in jejunal tissues (*n* = 5). (E) Representative colon histology images by H&E staining and TEM. (F) Relative mRNA levels of *ZO‐1* and *Occludin* in colon tissues (*n* = 4). (G) Experimental design from *B. subtilis* supernatant cocultured with IPEC‐J2 cells. (H) Western blot analysis of GAPDH, Occludin, and ZO‐1 in IPEC‐J2 cells. (I) Experimental design for investigating the effects of *B. subtilis* supernatant treatment in LPS‐induced damage in IPEC‐J2 cells. (J) Western blot analysis of GAPDH, Occludin, and ZO‐1. Statistical analysis was performed using an ANOVA followed by Fisher's least significant difference test. The data are shown as means ± SEM. BS.sup, *B. subtilis* supernatant; HI BS, heat‐inactivated *B. subtilis*; LB, Luria‐Bertani culture medium.

To investigate the beneficial effect of *B. subtilis*, wild type (WT) mice were gavaged with heat‐inactivated *B. subtilis* (HI BS) or filtered *B. subtilis* supernatant (BS. sup) and then subjected to treatment with LPS (Figure 2A). Such a pretreatment with BS.sup, but not HI BS, significantly decreased inflammatory cytokine levels in the serum, as visible through TNF‐α and IL‐6 levels (Figure 2B). Both BS.sup and HI BS treatment reduced the levels of serum IL‐1β (Figure 2B). Histological analyses showed that LPS induced atrophy of intestinal villi and thickening of the basal layer, while adversely, BS.sup treatment restored morphology of the intestinal villi and the protein expression of Occludin (Figure 2C). Indeed, BS. sup treatment increased mRNA expression of *ZO‐1* and *Occludin* compared to the LPS group (Figure 2D). Additionally, both BS. sup and HI BS treatment reduced mRNA expression of *Tnfα* in the jejunum (Figure S3A). In colon tissues, the results of hematoxylin and eosin (H&E) staining and transmission electron microscopy indicated that LPS also caused atrophy and damage to the intestinal villi, while BS.sup treatment protected intestinal morphology (Figure 2E). As evident from mRNA levels, both BS.sup and HI BS treatment rescued the levels of *ZO‐1* and *Occludin* (Figure 2F). Compared to the HI BS group, BS. sup treatment significantly increased the levels of *ZO‐1* and *Occludin* (Figure 2F).

To further compare the effects of BS.sup and HI BS on intestinal tight junction proteins, we treated IPEC‐J2 cells with BS.sup or HI BS for 24 h in vitro (Figure 2G). Compared with the control group (0%), supplementing 10% BS.sup significantly increased the expression of ZO‐1 and Occludin (Figure 2H, S3B). Supplementing 5% BS.sup also significantly upregulated the protein levels of Occludin (Figure 2H). In addition, exposure to HI BS also increased the expression of Occludin, while decreasing the expression of ZO‐1 (Figures S3C, 3D). In the LPS‐induced intestinal barrier injury model, the expression of intestinal tight junction proteins in IPEC‐J2 cells was significantly decreased by LPS treatment (Figure 2I,J). When pre‐treating with 10% BS.sup, we found that the protein expression of ZO‐1 and Occludin was restored (Figure 2J). Thus, the protective effects of *B. subtilis* against LPS‐induced acute intestinal injury are primarily associated with its metabolites.

### Effects of *B. subtilis* on the gut metabolic profile and the identification of key metabolites derived from *B. subtilis*

**Figure 3:**
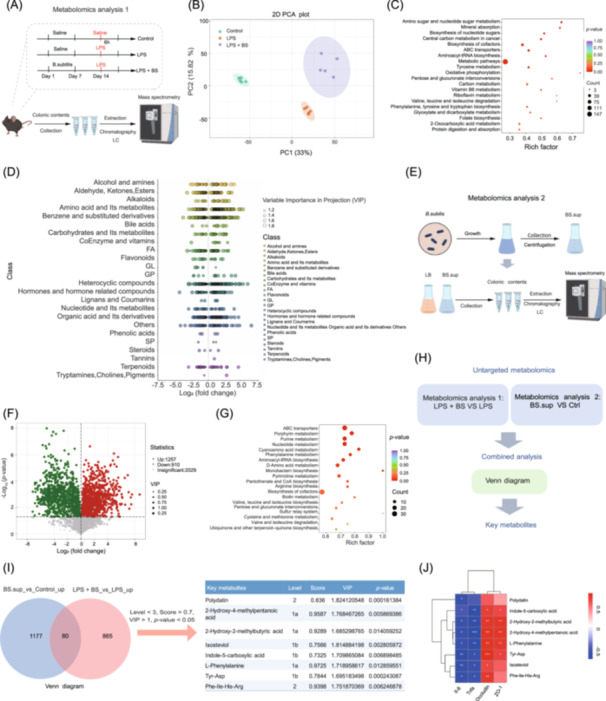
Identification of key metabolites secreted by *B. subtilis*. (A) Metabolomics of colon contents from mouse experiment 1 (*n* = 5). (B) A principal component analysis (PCA) plot for colon metabolomics in the Control, LPS, and LPS + BS groups. (C) Enrichment analysis of significantly differentially secreted metabolites. (D) Distribution of the metabolites at the class level. (E) Metabolomics of LB culture medium and supernatant derived from *B. subtilis* (*n* = 3). (F) A volcano plot showing the differentially secreted metabolites (VIP > 1, *p*‐value < 0.05). (G) Enrichment analysis of significantly differentially secreted metabolites. (H) The process of a combined analysis to identify key metabolites. (I) Screened core metabolites. (J) Correlation analysis between key metabolites and the expression levels of tight junction proteins and inflammatory factors. Ctrl, control; LB, Luria‐Bertani.

To uncover the metabolites that modulate gut barrier function, we first performed untargeted metabolomic analysis on colonic contents samples collected from control, LPS treatment (LPS), and LPS + BS groups (mouse experiment 1, Figure 3A). Principal component analysis suggested that samples were clustered according to different treatments, thus indicating that the treatments altered metabolite composition (Figure 3B). To identify the changes in metabolite profiles among the three groups, these differential metabolites were screened by variable importance in projection (VIP) > 1 and *p*‐value < 0.05 (ANOVA). KEGG analysis suggested that significantly different metabolites were mainly due to metabolic pathways, such as tyrosine metabolism, carbon metabolism, and vitamin B6 metabolism (Figure 3C). To visualize the types of different metabolites at the class level between the LPS + BS group and the LPS group, we also created a bubble plot (Figure 3D). We observed a large number of different metabolites related to amino acid metabolism and its metabolites, heterocyclic compounds, and organic acids and their derivatives (Figure 3D). Examining the culture medium supernatant of *B. subtilis* and LB control culture medium using untargeted metabolome analyses (Figure 3E), we found 1257 significantly upregulated metabolites and 910 significantly downregulated metabolites in the culture medium supernatant of *B. subtilis* as shown in a volcano plot (Figure 3F). Again, a large number of metabolites are related to amino acids and their metabolites (Figure S4A,B). Notably, *B. subtilis* also produced short‐chain fatty acids, such as isobutyric acid, valeric acid, and glutaric acid (Figure S4C). In addition, KEGG analysis of differentially expressed metabolites suggested that ABC transporters, purine, and nucleotide metabolism were among the enriched pathways (Figure 3G).

To examine the key metabolites mediating the effect of *B. subtilis*, we performed a joint analysis combining colon metabolic profiles and the culture medium supernatant of *B. subtilis* (Figure 3H). We identified 80 overlapping metabolites by joint analysis of “LPS + BS_VS_LPS_up (in vivo experiment)” and “BS.sup_vs_control_up (in vitro experiment)” (Figure 3I). To find out the potential key functional metabolites, we further screened based on the following conditions: matching level < 3, matching score > 0.7, VIP value > 1.6, and *p*‐value < 0.05 (Figure 3I). Finally, we identified eight metabolites, including HMP, 2‐hydroxy‐2‐methylbutyric acid (HMBA), polydatin, indole‐5‐carboxylic acid (ICA), isosteviol, l‐phenylalanine (l‐Phe), Tyr‐Asp, and Phe‐Ile‐His‐Arg (Figure 3I). Moreover, correlation analysis results indicate that the abundance of the eight metabolites was correlated with the expression of ZO‐1 and Occludin (Figure 3J).

### Metabolite HMP, derived from *B. subtilis*, improves the expression of tight junction protein and intestinal barrier integrity

**Figure 4:**
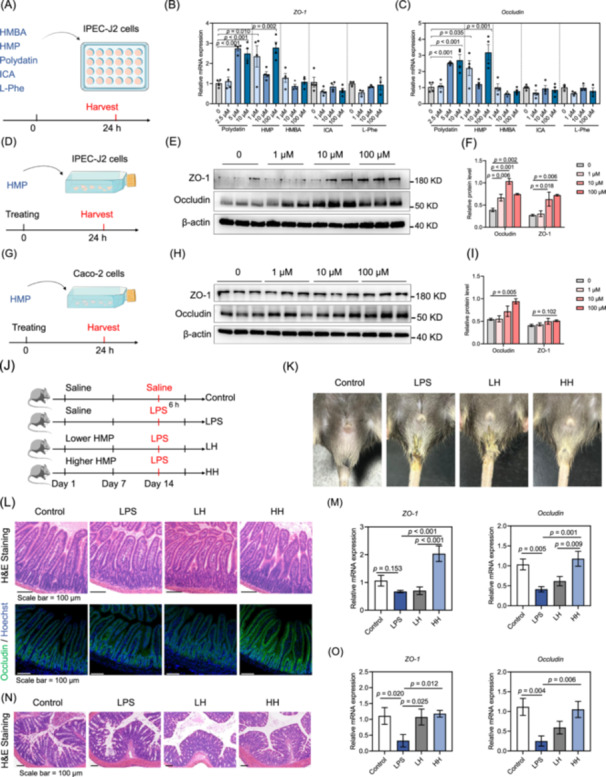
HMP derived from *B. subtilis* improves intestinal epithelial barrier in vivo and in vitro. (A) Experimental design of the metabolites exposed to IPEC‐J2 cells. (B–C) Relative mRNA levels of *ZO‐1* and *Occludin*, and (*n* = 4). (D) Experimental design of HMP exposed to IPEC‐J2 cells. (E) Western blot analysis of ZO‐1 and Occludin (*n* = 3). (F) Relative protein levels were normalized to those of control β‐actin. (G) Experimental design of HMP exposed to Caco‐2 cells. (H) Western blot analysis of Occludin and ZO‐1 (*n* = 3). (I) Relative protein levels were normalized to those of control β‐actin. (J) Experimental design of the mouse model. (K) Representative images of diarrhea symptoms. (L) Representative jejunal histology images by H&E staining and immunofluorescence of Occludin. (M) Relative mRNA levels of *ZO‐1* and *Occludin* from jejunal tissues (*n* = 5). (N) Representative colon histology images by H&E staining. (O) Relative mRNA levels of *ZO‐1* and *Occludin* from colon tissues (*n* = 5). Statistical analysis was performed by one‐way analysis of variance (ANOVA) followed by Fisher's least significant difference procedure. Data are represented as means ± SEM. HMBA, 2‐hydroxy‐2‐methylbutyric acid; HMP, 2‐hydroxy‐4‐methylpentanoic acid; HH, high‐dose HMP; ICA, indole‐5‐carboxylic acid; LH, low‐dose HMP; l‐Phe, l‐phenylalanine.

To investigate the effects of metabolites derived from *B. subtilis* on the intestinal barrier, we treated IPEC‐J2 cells with 5 metabolites (HMP, HMBA, polydatin, ICA, and l‐Phe) for 24 h (Figure 4A). HMP and polydatin significantly increased mRNA expression of *ZO‐1* and *Occludin*, while HMBA, ICA, and l‐Phe did not affect the expression of intestinal tight junction protein (Figure 4B,C). Next, we investigated the effects of HMP and polydatin on the expression of tight junction protein in IPEC‐J2 cells (Figure 4D, Figure S5A). Treating with 1, 10, or 100 μM HMP significantly increased protein levels of ZO‐1 and Occludin (Figure 4E,F). However, polydatin had no significant effects on the protein levels of Occludin and ZO‐1 (Figure S5B,C). In addition, we also explored the effects of HMP on protein expression of tight junction protein in Caco‐2 cells (Figure 4G). Indeed, a 100 μM HMP treatment significantly increased the protein levels of Occludin (Figure 4H,I).

Next, we investigated the effects of HMP in vivo. Eight‐week‐old male C57BL/6J mice were gavaged with sterile saline or HMP for 14 days, followed by intraperitoneal injection of LPS on day 14 (Figure 4J). LPS treatment induced a diarrhea phenotype, while mice in the LPS + high HMP or LPS + low HMP group showed less severe diarrhea symptoms (Figure 4K). Histological analyses suggested that LPS caused atrophy in the intestinal villi of jejunum, and HMP treatment maintained the intestinal villi morphology during LPS stimulation (Figure 4L). As for mRNA levels, LPS treatment decreased the expression of *Occludin* and *ZO‐1*, and high‐HMP treatment restored the levels of *Occludin* and *ZO‐1* (Figure 4M). High‐HMP treatment also rescued the protein levels of Occludin (Figure S5D,E). Additionally, LPS treatment damaged the morphology of colon tissue, while HMP treatment protected intestinal morphology and maintained the levels of *Occludin* and *ZO‐1* (Figure 4N–O). Our data suggest that HMP was responsible for the protective effect of *B. subtilis* on the intestinal barrier.

### GADD45A is a novel regulator of the intestinal epithelial barrier

To identify potential regulators associated with the maintenance of intestinal barrier integrity, we re‐analyzed publicly accessible RNA sequencing data. We observed that the expression of intestinal tight junction proteins was gradually reduced in the DSS colitis mouse model and that expression levels of the GADD45 family decreased in parallel (Figure S6A). In colonic samples of both healthy individuals and those afflicted with ulcerative colitis (UC), we found that GADD45A was significantly decreased in patients with UC (Figure S6B). Similarly, we compared the publicly available data derived from Caco‐2 cells, discovering that ochratoxin A treatment significantly reduced the expression of tight junction proteins ZO‐1 and Occludin, as well as the expression levels of Gadd45 family members, such as *Gadd45a*, *Gadd45b*, and *Gadd45g* (Figure S6C). Thus, the Gadd45 family, particularly *Gadd45a*, may play a significant role in regulating intestinal inflammation and intestinal barrier integrity. Indeed, a recent study demonstrated that GADD45A is involved in regulating the maintenance and function of intestinal stem cells (ISCs) [28].

**Figure 5:**
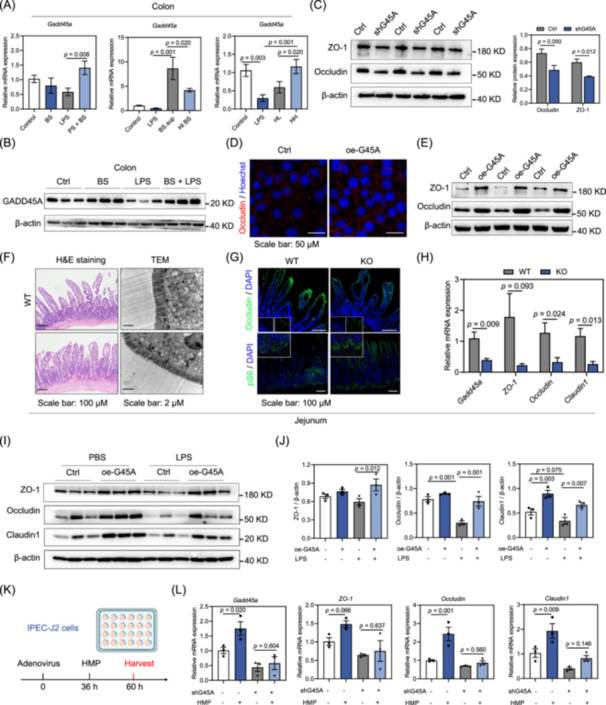
GADD45A is a novel regulator of the intestinal epithelial barrier. (A) Relative mRNA levels of *Gadd45a* in colon tissues (*n* = 5). (B) Western blot analysis of GADD45A in colon tissues (*n* = 3). (C) Western blot analysis of ZO‐1, Occludin, and β‐actin (*n* = 3). (D) Representative immunofluorescence of Occludin in IPEC‐J2 cells. (E) Western blot analysis of ZO‐1, Occludin, and β‐actin in WT and oe‐G45A cells (*n* = 3). (F) Representative jejunal histology images by H&E staining and transmission electron microscopy. (G) Immunofluorescence of Occludin, pS6, and DAPI. (H) Relative mRNA levels of *Gadd45a* and intestinal tight junction proteins in WT and KO mice (*n* = 6). (I) After supplementing control and oe‐G45A *adenovirus* for 36 h, the cells were treated with PBS or 10 μg/mL LPS for 24 h. Western blot analysis of ZO‐1, Occludin, and β‐actin. (J) Relative protein levels were normalized to those of control β‐actin. (K) After supplementing control and shG45A *adenovirus* for 36 h, cells were treated with PBS or 100 μM HMP for 24 h. (L) Relative mRNA levels of *Gadd45a*, *Occludin*, *ZO‐1*, and *Claudin1* (*n* = 3). shG45A, knockdown of *Gadd45a*; oe‐G45A, overexpression of *Gadd45a*; pS6, S6 ribosomal protein phosphorylation.

Next, we examined the effects of *B. subtilis* and HMP on the expression of GADD45A. We observed that *B. subtilis* or HMP treatment rescued mRNA levels of *Gadd45a* in LPS‐induced acute intestinal injury models (Figure 5A, Figure S6D). Consistently, compared to the LPS group, *B. subtilis* treatment significantly increased the levels of GADD45A protein in the jejunal and colonic tissues (Figure 5B, Figure S6E, F). HMP treatment also restored protein expression of GADD45A in LPS‐induced acute intestinal injury (Figure S6G,H).

To assess the effect of GADD45A on intestinal tight junction protein and intestinal barrier integrity, we conducted gain‐of‐function and loss‐of‐function experiments in IPEC‐J2 cells. Compared with the control (Ctrl) cells, the knockdown of Gadd45a (shG45A) significantly decreased the expression of Occludin and ZO‐1 (Figure 5C). In contrast, Gadd45a overexpression (oe‐G45A) increased the expression levels of Occludin and ZO‐1 in IPEC‐J2 cells (Figure 5D,E). GADD45A might thus improve intestinal tight junction protein expression and intestinal barrier function.

### GADD45a is a key mediator of the protective effects of *B. subtilis* against acute intestinal injury

We further investigated GADD45A and its effect on intestinal barrier integrity in vivo and generated Gadd45a knockout (KO) mice. Gadd45a deficiency leads to a decrease in intestinal villus height, as evidenced by H&E staining and TEM (Figure 5F, Figure S7A). Gadd45a deficiency also caused a reduction in Occludin expression according to the immunofluorescence results (Figure 5G). mTOR hyperactivation induced necroptosis of the epithelium, disruption of the intestinal barrier, and sensitivity for DSS‐induced colitis [29]. Levels of S6 ribosomal protein phosphorylation (pS6) were increased in the jejunum of Gadd45a KO mice (Figure 5G, Figure S7B). We also observed that Gadd45a deficiency significantly decreased mRNA expression of genes related to the intestinal tight junction, such as *Occludin* and *ZO‐1* (Figure 5H). Gadd45a deficiency thus might decrease intestinal tight junction proteins.

Next, we knocked down GADD45A in IPEC‐J2 cells using adenovirus and then treated them with LPS to better understand GADD45A deficiency during LPS‐induced acute intestinal injury. shG45a significantly decreased the expression of ZO‐1, Occludin, and Claudin1 (Figure S7C,D). LPS decreased intestinal tight junction proteins, while oe‐G45A restored the levels of ZO‐1, Occludin, and Claudin1 in IPEC‐J2 cells (Figure 5I,J). Together, oe‐G45A maintained the integrity of the intestinal barrier against LPS‐induced injury.

We further investigated whether HMP increased the expression of intestinal tight junction proteins in a GADD45A‐dependent manner (Figure 5K). At the mRNA level, HMP did not maintain the expression of tight junction proteins in the absence of GADD45A (Figure 5L). We demonstrate that the enhancement of intestinal barrier function by HMP was dependent on GADD45A.

### GADD45A enhances intestinal barrier function via Wnt/β‐catenin pathway

When exploring the downstream pathways of GADD45A for enhancing intestinal barrier function, we performed RNA‐seq analysis on IPEC‐J2 cells from the control (Ctrl) group and the oe‐G45A group. Overexpression of GADD45A had significantly altered mRNA expression profiles according to the heatmap (Figure S8A). Compared to the Ctrl group, 528 genes were significantly upregulated, and 240 genes were significantly downregulated in the oe‐G45A group as demonstrated by the volcano plot (Figure S8B). KEGG analysis using the significantly upregulated genes suggested that the intestinal immune system, toll‐like receptor signaling, ECM‐receptor interaction, and p53 signaling pathways were enriched (Figure S8C). In addition, KEGG analysis run on the significantly downregulated genes showed that Gap junction, Hippo signaling, and the Wnt signaling pathways were enriched (Figure S8D). Notably, the heatmap showing the expression of genes in the Wnt signaling pathway suggested that oe‐G45A affected Wnt signaling, indicating a potential regulatory role in this pathway (Figure S8E). Additionally, oe‐G45A had significantly altered gene expression relating to the mTOR signaling pathway (Figure S8F). Consistent with the RNA‐seq analysis, we found that oe‐G45A significantly increased the expression of some key factors in Wnt signaling pathway, such as *DVL2* and *DVL3*, by qPCR analysis (Figure S8G). Consistently, shG45A significantly decreased β‐catenin protein phosphorylation and increased S6 ribosomal protein phosphorylation (Figure S8H). Wnt/β‐catenin and mTOR signaling pathways may be downstream of GADD45A when regulating intestinal barrier homeostasis.

To verify whether GADD45A improved intestinal epithelial barrier dependent on the Wnt/β‐catenin signaling pathway, IPEC‐J2 cells were treated with control or shG45A *adenovirus* for 36 h and then cultivated with a Wnt agonist BML‐284 (Figure S8I). We found that BML‐284 rescued the downregulation of *Occludin*, *ZO‐1*, *ZO‐2*, and *Claudin1* induced by shG45A (Figure S8J). At the protein level, shG45a decreased the protein levels of ZO‐1, while BML‐284 treatment rescued the expression of ZO‐1 (Figure S8K). Consistently, a Wnt antagonist, LF3, blocked the GADD45A‐induced increase in ZO‐1 protein expression (Figure S8L). Taken together, our data suggest that GADD45A improved the intestinal epithelial barrier dependent on the Wnt/β‐catenin signaling pathway.

## DISCUSSION

Intestinal barrier integrity is fundamental to intestinal health, and its dysregulation relates to the pathophysiology of numerous gastrointestinal diseases [30]. Preservation and restoration of this barrier are key targets in the management of gastrointestinal disorders. Here, we show that the probiotic *B. subtilis* alleviates acute intestinal injury and mucosal barrier dysfunction in an LPS‐induced acute intestinal injury mouse model. The effect of *B. subtilis* is primarily mediated through its metabolites. In the present study, we observed that a novel metabolite secreted by *B. subtilis*, HMP, enhanced the integrity of the intestinal barrier by activating GADD45A. Furthermore, we investigated the mechanism through which GADD45A affected the intestinal barrier function.

Recently, many studies have reported the effects of *B. subtilis*, such as inhibiting *Salmonella* infection [31], maintaining gut microbiota homeostasis [32], alleviating intestinal oxidative injury [33], and improving intestinal integrity [34]. In parallel, the efficacy and safety of *B. subtilis* were found to be promising in clinical trials [20, 24, 26]. Consistent with previous studies [35, 36], our results demonstrate that *B. subtilis* ameliorated an LPS‐induced intestinal epithelial barrier dysfunction in mice. Although the role of *B. subtilis* in maintaining intestinal homeostasis and health is well recognized, the detailed mechanisms of action remain poorly understood, thereby limiting its therapeutic application. Few studies addressed the effective functional components derived from *B. subtilis*, such as metabolites and antimicrobial peptides, as well as their underlying mechanism [37, 38, 39]. Leistikow et al. found that *B. subtilis*‐derived peptides combated multidrug‐resistant *S. aureus* infections and improved antibiotic efficacy by disrupting its quorum sensing and biofilm assembly [37]. We thus compared the regulatory effects of BS.sup and HI BS on the intestinal barrier and discovered that metabolites derived from *B. subtilis* played a prominent role in regulating the integrity of the intestinal epithelial barrier through in vivo and in vitro experiments.

Gut microbiota also plays an indispensable role in the regulation of the homeostasis of the host's intestinal epithelial barrier [40]. We also observed that oral administration of *B. subtilis* altered the composition of gut microbiota. Specifically, *B. subtilis* affected the abundance of *Akkermansia* and *Limosilactobacillus*, which is strongly and positively correlated with intestinal tight junction protein. Some species have been found to regulate the function of the intestinal barrier [41]. *Akkermansia muciniphila* has been shown to accelerate ISC‐mediated epithelial development and protect the intestine from irradiation‐induced injury by secreting propionic acid [41, 42]. Thus, the crosstalk between *B. subtilis* and host microbiota, particularly *Akkermansia*, might also relate to intestinal barrier integrity.

Our present study identified a novel bioactive metabolite, HMP, derived from *B. subtilis*, and demonstrated its beneficial role. To date, few studies are available on the physiological functions of HMP. Recently, it was reported that either the colonization of *R. torques* or oral administration of HMP intervention alleviated inflammation and fibrosis in a metabolic dysfunction‐associated steatohepatitis mouse model [43]. We demonstrate that HMP enhanced intestinal barrier function by upregulating the expression of GADD45A in intestinal epithelial cells. However, how precisely HMP‐activated GADD45A was not explored in our study, and the presence of an HMP receptor in intestinal epithelial cells would be an interesting question to pursue. In addition to HMP, *B. subtilis*, also secretes other metabolites, and it remains to be further demonstrated whether HMP is necessary for the beneficial effects of *B. subtilis* on intestinal barrier integrity. Also, knowing the enzymes in *B. subtilis* responsible for the synthesis of HMP and their biosynthetic pathways is a necessary next step. The regulatory effect of a combined application of *B. subtilis* and HMP on intestinal epithelial barrier integrity requires further investigation.

GADD45A is a histone‐folding protein controlled by p53 induced by various cellular stresses [44]. Our previous studies have shown that GADD45A regulates lipid infiltration in skeletal muscle and the development of brown fat, playing an important role in lipid metabolism homeostasis [44, 45]. Several studies have indicated that GADD45A may serve as a key target for treating intestinal diseases. For example, the expression of GADD45A in tumor tissue from colorectal cancer patients was significantly downregulated compared with normal mucosa tissue [46]. However, it is not yet known whether GADD45A is involved in regulating intestinal barrier integrity. Results from this study demonstrate that GADD45A deficiency decreased the expression of intestinal tight junction proteins both in vivo and in vitro. oe‐G45A improved intestinal barrier function and protected intestinal barrier integrity from LPS stimulation. GADD45A enhanced the expression of intestinal tight junction proteins dependent on the Wnt/β‐catenin signaling pathway. Notably, the molecular mechanism by which GADD45A activated the Wnt/β‐catenin pathway remains to be investigated.

β‐catenin‐mediated canonical Wnt pathway regulates intestinal cell proliferation, differentiation, and homeostasis of intestinal epithelia [47, 48]. Using a Wnt signaling activator [49], BML‐284 did not affect the expression of intestinal tight junction proteins, while BML‐284 could restore tight junction protein expression caused by knocking down GADD45A in IPEC‐J2 cells. The enhanced effect of GADD45A on tight junction protein expression was mediated by the Wnt signaling pathway. However, we have not yet investigated whether the BML‐284 treatment similarly restored the expression levels of tight junction proteins and maintained the integrity of the intestinal barrier in GADD45A‐deficient mice.

## CONCLUSION

**Figure 6:**
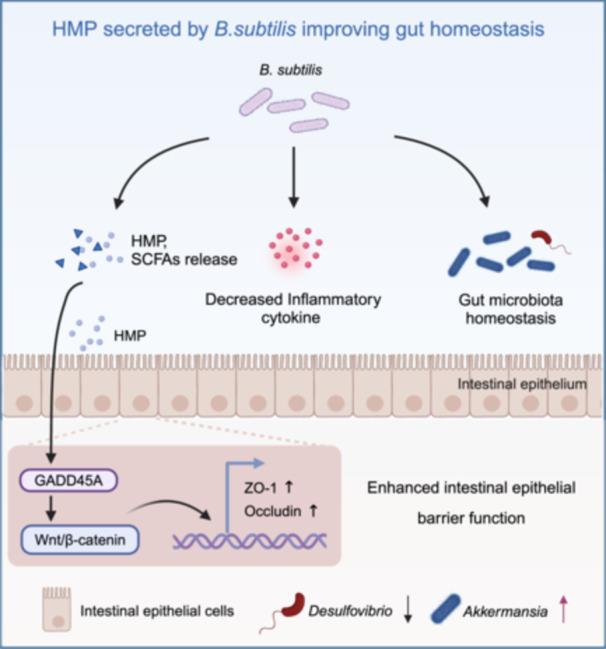
*B. subtilis* and its metabolite HMP alleviate LPS‐induced intestinal epithelial barrier damage via the GADD45A‐Wnt/β‐catenin axis. LPS leads to a significant disruption of gut homeostasis within a short time, visible by inflammatory responses, microbiota dysregulation, and intestinal barrier damage. *B. subtilis* administration could restore gut homeostasis by alleviating inflammatory responses, increasing the abundance of beneficial bacteria, and enhancing the intestinal epithelial barrier. The diagram was created using Microsoft PowerPoint and BioRender.com.

In summary, our study addressing the beneficial effects of *B. subtilis* on the intestinal barrier during LPS‐induced injury and inflammation, pointed to its metabolites playing a primary role (Figure 6). We show for the first time that HMP derived from *B. subtilis* regulates intestinal barrier function. In addition, we identified GADD45A as a novel regulator involved in intestinal barrier integrity and the development of ulcerative colitis. GADD45A improves the expression of intestinal tight junction proteins and maintains intestinal barrier integrity via its downstream Wnt/β‐catenin pathway. Furthermore, HMP can improve intestinal barrier integrity, which is dependent on GADD45A. Our study provides the basis for developing probiotics as therapeutic approaches towards clinically relevant intestinal diseases.

## METHODS

### Animals

Eight‐week‐old male wild‐type (WT) C57BL/6J mice were purchased from GemPharmatech Co., Ltd (Jiangsu, China). Gadd45a‐knockout (Gadd45a^−/−^) mice [50] were generously provided by Professor Albert J. Fornace Jr. (Gene Response Section, DBS, National Cancer Institute, USA) and maintained on a C57BL/6 genetic background. Both Gadd45a^−/−^ mice and their wild‐type littermate controls, serving as controls, were derived from the vital breeding colony of Gadd45a heterozygous mice maintained at Hangzhou Normal University. All mice were housed under specific pathogen‐free conditions, with free unlimited access to water and standard rodent chow food.

To investigate the effects of *B. subtilis* on LPS‐induced acute intestinal injury and inflammation, mice were gavaged with 200 μL sterile saline or 200 μL *B. subtilis* (1 × 10^9^ CFU/mL) for 14 days. Then, mice were intraperitoneally injected with LPS (0.1 mg/kg in sterile saline) or sterile saline. After being treated with LPS for 6 h, mice were humanely euthanized.

To examine the protective component of *B. subtilis*, mice were gavaged with 200 μL Luria‐Bertani (LB) culture medium, heat‐inactivated (HI) *B. subtilis*, or filtered *B. subtilis* supernatant (BS.sup) for 14 days and then subjected to LPS treatment for 6 h. HI *B. subtilis* and filtered BS.sup were collected, with the *B. subtilis* concentration reaching 1 × 10^9^ CFU/mL.

To investigate the effects of HMP derived from *B. subtilis*, mice were gavaged with 200 μL sterile saline, 100 mg/kg BW HMP (low‐dose HMP, LH), or 200 mg/kg BW HMP (high‐dose HMP, HH) for 14 days and then subjected to LPS treatment for 6 h. HMP was purchased from Energy Chemical (A01045455‐5G) and dissolved in sterile saline.

To investigate the effects of *B. subtilis*‐derived FF on the intestinal barrier function of pigs, the diets of pigs were supplemented with 0%, 5%, and 10% of *B. subtilis*‐derived FF, respectively. The protein levels of the three diets were adjusted to be consistent, and the substrate for the FF consisted of corn, soybean meal, and bran. The pre‐feeding period lasted 4 days, and the experimental period lasted 38 days.

### Bacterial stains

*B. subtilis* was cultured in LB culture medium, containing 10 g/L tryptone, yeast extract 5 g/L, and 10 g/L NaCl. *B. subtilis* is cultured in a constant temperature shaker at 37°C with a rotation speed of 180 rpm/min.

### Cell culture and LPS treatment

The IPEC‐J2 and Caco‐2 cell lines used in this study were provided by Shan Lab. The IPEC‐J2 and Caco‐2 cells were induced with culture medium containing Dulbecco's modification of Eagle's medium, 10% FBS (Gibco), and 1% penicillin/streptomycin at 37°C with 5% CO_2_, followed by feeding with fresh medium every 2 days. 10 mg/L LPS purchased from MCE was supplemented in culture medium for 24 h to induce intestinal epithelial injury.

### Measurements of serum TNF‐α, IL‐6, and IL‐1β

The levels of serum TNF‐α, IL‐6, and IL‐1β were measured using enzyme‐linked immunosorbent assay (ELISA) kits according to the manufacturer's instructions and analyzed in a microplate reader at 450 nm. The ELISA kits were purchased from Jiangsu Meimian Industrial Co., Ltd.

### Histopathological analysis

Jejunum and colon samples soaked in 4% paraformaldehyde solution were dehydrated, embedded in paraffin, and stained with haematoxylin and eosin, as described in our previous study [51].

### Immunofluorescence

Immunofluorescence staining was performed as previously described [44]. Fluorescent images were captured as single‐channel grayscale images using a Leica DM 6000B fluorescent microscope with a ×20 objective (NA 0.70). Antibodies are detailed in Table S1.

### Western blot

The western blot analysis was performed as described previously [44, 52]. The primary antibodies are presented in Table S1. Immunodetection was performed using an enhanced chemiluminescence western blot analysis substrate (BL523A, Biosharp, Beijing, China) and detected with a ChemiScope 6200 Western Imaging Analyzer (Clinx Science Instruments Co., Ltd).

### High‐throughput 16S rRNA gene amplicon sequencing and analysis

Sequence analyses were conducted by Uparse software (version 7.0.1001, http://drive5.com/uparse/) [53]. Sequences exhibiting ≥97% homology were clustered into the same operational taxonomic units (OTUs). A consensus sequence for each OTU was selected for subsequent annotation. Abundance out data was standardized by referencing the sample with the minimum sequence count and ensuring that alpha diversity and beta diversity were accounted for by utilizing these normalized datasets. Beta diversity analysis was used to investigate the differences in species composition between samples. PCoA was employed to extract the principal coordinates, providing a visual synopsis of the intricate, multidimensional data set. The visualization of the PCoA results was facilitated by the stats and ggplot2 packages in the R programming environment (version 3.6.3).

### Metabolomics

For multi‐group analyses, differential metabolites were identified based on the VIP score (VIP > 1) and *p*‐value (*p* < 0.05, ANOVA). The VIP score was extracted from the results of the Orthogonal Projections to Latent Structures Discriminant Analysis (OPLS‐DA), which also included score plots and permutation plots. These results were generated using the R package (version 3.6.3). Before OPLS‐DA, the data underwent logarithmic transformation (log_2_) and centering of means. To prevent overfitting, a permutation test was conducted with 200 permutations. The identified metabolites were annotated using the KEGG Compound Database (http://www.kegg.jp/kegg/compound/), and subsequently mapped onto the KEGG Pathway Database (http://www.kegg.jp/kegg/pathway.html) for pathway analysis.

### Screening of key metabolites derived from *B. subtilis*

First, we did a joint analysis of the significantly changed metabolites of the “LPS + BS_VS_LPS_up (in vivo experiment)” and the “BS.sup_vs_Control_up (in vitro experiment)” to find the overlapping differential metabolites. Then, we screened the key metabolites based on the following conditions: matching level < 3, Score > 0.7, VIP value > 1.6, and *p*‐value < 0.05. In addition, correlation analysis between the abundance of the identified metabolites and the expression of tight junction proteins was also used to identify the key metabolites.

### Real‐time quantitative polymerase chain reaction (qPCR) analysis

Detailed experimental materials and procedures of RNA extraction, library construction, and qPCR were available in the Supplementary Material. The gene‐specific primer sequences are listed in Table S2. Relative gene expression was analyzed using the 2^−ΔΔCT^ method.

### Statistical analysis

Statistical analyses were carried out in SPSS, version 25.0 (IBM Corporation), and R (version 3.6.3). Our results are visualized by GraphPad Prism 8.4 software (Prism Inc.), and all data are given as means ± standard error of measurement. Data comparisons between the two groups were done using a two‐tailed unpaired Student's *t*‐test. Multivariate data were analyzed using one‐way ANOVA coupled with the least significant difference multiple comparisons. Differences are considered statistically significant at *p*‐value < 0.05. Detailed experimental materials and procedures, including sample collection and processing techniques and statistical analysis approaches, are available in the Supplementary Material.

## AUTHOR CONTRIBUTIONS

**Shiqi Liu**: Writing—original draft; investigation; data curation; visualization; software; validation. **Peiran Cai**: Data curation; investigation. **Wenjing You**: Investigation; data curation. **Mingshun Yang**: Investigation. **Yuang Tu**: Investigation. **Yanbing Zhou**: Investigation. **Teresa G Valencak**: Writing—review and editing. **Yingping Xiao**: Writing—review and editing; investigation. **Yizhen Wang**: Supervision; formal analysis. **Tizhong Shan**: Supervision; funding acquisition; conceptualization; methodology.

## CONFLICT OF INTEREST STATEMENT

The authors declare no conflicts of interest.

## ETHICS STATEMENT

The experimental protocol and procedures for the care and treatment of the mice were approved (No. ZJU20240229) by the Zhejiang University Animal Care and Use Committee.

## Supporting information


**Figure S1.** *B. subtilis* alleviates intestinal inflammation and improves antioxidant capacity.
**Figure S2.** *B. subtilis* alters gut microbial composition and increases the abundance of beneficial bacteria.
**Figure S3.** Enhancement of intestinal epithelial barrier function by *B. subtilis* was primarily associated with metabolites.
**Figure S4.** Analysis of metabolites derived from *B. subtilis*.
**Figure S5.** Effects of metabolites derived from *B. subtilis* on intestinal tight junction proteins.
**Figure S6.** Gadd45A is a key regulator of intestinal barrier integrity.
**Figure S7.** Effects of GADD45A deficiency on intestinal epithelial barrier.
**Figure S8.** GADD45A enhanced intestinal epithelial barrier dependent on Wnt/β‐catenin signaling pathway.

**Table S1.** Antibodies for western blots or immunofluorescence.
**Table S2.** Information of primers.

## Data Availability

The metabolomics data reported in this paper have been deposited in the OMIX, China National Center for Bioinformation/Beijing Institute of Genomics, Chinese Academy of Sciences (https://ngdc.cncb.ac.cn/omix/select-edit/OMIX008263 and https://ngdc.cncb.ac.cn/omix/select-edit/OMIX008265). The high‐throughput 16S rRNA gene amplicon sequencing data reported in this paper have been deposited in the Genome Sequence Archive at the National Genomics Data Center, China National Center for Bioinformation/Beijing Institute of Genomics, Chinese Academy of Sciences (https://ngdc.cncb.ac.cn/gsa/search?searchTerm=CRA021310). The data and scripts for analysis and visualization are saved in GitHub https://github.com/lsqaa/iMeta. Supplementary materials (methods, figures, tables, graphical abstract, slides, videos, Chinese translated version, and update materials) may be found in the online DOI or iMeta Science http://www.imeta.science/.
