# Supplementary material for: Enhancement of gut barrier integrity by a Bacillus subtilis secreted metabolite through the GADD45A‐Wnt/β‐catenin pathway
Source: Imeta. 2025 Feb 23;4(2):e70005. doi: 10.1002/imt2.70005 (PMC11995189; doi:10.1002/imt2.70005)
Supplement: Supplementary file 1 — Figure S1. B. subtilis alleviates intestinal inflammation and improves antioxidant capacity. Figure S2. B. subtilis alters gut microbial composition and increases the abundance of beneficial bacteria. Figure S3. Enhancement of intestinal epithelial barrier function by B. subtilis was primarily associated with metabolites. Figure S4. Analysis of metabolites derived from B. subtilis. Figure S5. Effects of metabolites derived from B. subtilis on intestinal tight junction proteins. Figure S6. Gadd45A is a key regulator of intestinal barrier integrity. Figure S7. Effects of GADD45A deficiency on intestinal epithelial barrier. Figure S8. GADD45A enhanced intestinal epithelial barrier dependent on Wnt/β‐catenin signaling pathway. [file IMT2-4-e70005-s002.docx]

Supporting Information to

**Enhancement of gut barrier integrity by a *Bacillus subtilis* secreted metabolite through the GADD45A-Wnt/β-catenin pathway**

**Running title:** A probiotic metabolite improves integrity of intestinal epithelial barrier

Shiqi Liu^1,2,3^, Peiran Cai^1,2,3^, Wenjing You^1,2,3^, Mingshun Yang^1,2,3^, Yuang Tu^1,2,3^, Yanbing Zhou^1,2,3^, Teresa G. Valencak^1^, Yingping Xiao^4^, Yizhen Wang^1,2,3^, Tizhong Shan^1,2,3^*

^1^ College of Animal Sciences, Zhejiang University, Hangzhou 310058, China;

^2^ Key Laboratory of Molecular Animal Nutrition (Zhejiang University), Ministry of Education, Hangzhou 310058, China;

^3^ Zhejiang Key Laboratory of Nutrition and Breeding for High-quality Animal Products, Hangzhou 310058, China;

^4^ State Key Laboratory for Managing Biotic and Chemical Threats to the Quality and Safety of Agro-products, Institute of Agro-product Safety and Nutrition, Zhejiang Academy of Agricultural Sciences, Hangzhou 310021, China

* Corresponding author: [tzshan@zju.edu.cn](mailto:tzshan@zju.edu.cn) (Tizhong Shan)

**Supplementary methods**

**Immunoﬂuorescence**

Briefly, tissue samples were fixed with 4% paraformaldehyde (PFA) for 24 h and then embedded in paraffin to obtain the sections. The sections were immersed in xylene for deparaffinization and subsequently in a graded ethanol series for dehydration. Following this step, the antigens were retrieved using a commercial antigen retrieval kit (Servicebio, G1202-250ML, Wuhan, China). After that, sections were incubated with a blocking buffer containing 5% goat serum, 2% BSA, 0.2% Triton X-100, and 0.1% sodium azide in PBS for 1 h. Then, samples were incubated with primary antibodies overnight at 4℃. After washing with PBS three times, samples were incubated with secondary antibodies for 45 min at room temperature. Nuclei were exposed by incubating sections for 10 min with Hoechst 33258 (Beyotime, C1011, Shanghai, China).

**Real-time PCR analysis**

RNA extraction, library construction, and quantitative real-time PCR (qPCR) were performed as described in our previous study (Liu et al. 2023a). Total RNA was extracted from the samples using TRIzol reagent purchased from Yeasen Biotechnology (Shanghai) Co., Ltd. following the manufacturer’s instructions, and the purity and concentration of the total RNA were measured using a spectrophotometer (NanoDrop 2000; Thermo Fisher Scientific). A First Strand cDNA Synthesis Kit purchased from Yeasen Biotechnology (Shanghai) Co., Ltd. was used to generate cDNA by reverse transcription. qPCR was performed with an Applied Biosystems StepOnePlus Real-Time PCR System with SYBR Green Master Mix (Roche, Indianapolis, IN, United States). The gene-specific primer sequences are listed in Table S2. Relative gene expression was analyzed using the 2^-ΔΔCT^ method.

**Transmission electron microscopy (TEM) analysis**

The fresh colon samples were cut into small pieces (1 × 1 mm) and fixed with 2.5% glutaraldehyde overnight at 4°C. After washing them three times with phosphate buffer (0.1M, pH 7.0) for 15 minutes each time, the samples were fixed with 1% osmium tetroxide (in 0.1M pH 7.0 phosphate buffer) for 2 h at room temperature avoiding illumination. After being washed with a phosphate buffer (0.1M, pH 7.0), the samples designated for ultrastructural observation underwent a dehydration process. This involved sequential immersion in a graded ethanol series (30%, 50%, 70%, and 80%) followed by acetone solutions (90% and 95%), each for 15 minutes. The dehydration procedure was completed with two rounds of pure acetone for 20 min each. For infiltration, the specimen was initially immersed in a 1:1 mixture of pure acetone and the final Spurr resin solution for 1 h at room temperature. Subsequently, it was transferred to a 1:3 mixture of pure acetone and the same resin solution for 3 h and finally left to infiltrate the pure Spurr resin mixture overnight. The specimen was positioned in an Eppendorf tube containing Spurr resin and subjected to heating at 70°C for longer than 9 hrs. Following this, the specimen underwent sectioning using a LEICA EM UC7 ultramicrotome (Leica Microsystems, Wetzlar, Germany). The resulting sections were then stained with uranyl acetate and alkaline lead citrate for 5 to 10 min, respectively. The stained sections were analyzed utilizing a Hitachi Model H-7650 transmission electron microscope (TEM).

**Samples and libraries preparation of high-throughput 16S rRNA sequencing**

The colonic contents samples were freshly collected following the humane euthanasia of the mice and then immediately stored at – 80 °C. 16S rRNA sequencing was performed according to our previous study (Liu et al. 2023b). Sequencing libraries were constructed utilizing the TruSeq® DNA PCR-Free Sample Preparation Kit (Illumina, USA) by the protocol provided by the manufacturer, incorporating unique index codes. The quality of the libraries was evaluated using the Qubit@ 2.0 Fluorometer (Thermo Scientific) and the Agilent Bioanalyzer 2100 system. Subsequently, the libraries underwent sequencing on an Illumina NovaSeq platform, yielding 250 bp paired-end sequences.

**Metabolomics analyses**

The samples were stored at -80 °C before analysis. For sample preparation and extraction, take a 20 mg sample, add 400 μL extraction solution containing internal standard (methanol: water = 7:3, V/V), and vortex for 3 min. Subject the sample to ultrasonication in an ice bath for 10 min, vortex for an additional 1 min, and then place it at -20 °C for 30 min. Subsequently, the sample was centrifuged at 12,000 rpm for 10 min at 4°C. The supernatant was carefully collected and centrifuged at 12,000 rpm for 3 min. The supernatant was then transferred for subsequent liquid chromatography-mass spectrometry (LC-MS) analysis.

All samples were analyzed by two LC/MS methods. An aliquot of the sample was analyzed under positive ion conditions, utilizing a 0.1% aqueous formic acid solution as solvent A and a 0.1% formic acid in acetonitrile solution as solvent B for elution from a T3 column (Waters ACQUITY Premier HSS T3, 1.8 µm, 2.1 mm × 100 mm). The gradient elution program was as follows: an initial increase from 5% to 20% B over 2 min, followed by a ramp to 60% B within 3 min, a further increase to 99% B over 1 min, and maintenance at this percentage for 1.5 min. Subsequently, the column was re-equilibrated to 5% B over 0.1 min and held for an additional 2.4 min. The analytical conditions were set as follows: column temperature at 40°C, flow rate at 0.4 mL/min, and injection volume at 4 μL. An additional aliquot of the sample was analyzed under negative ion conditions, employing an identical elution gradient to that used in the positive mode.

The data collection was conducted using Analyst TF 1.7.1 software (Sciex, Concord, Ontario, Canada) in Information Dependent Acquisition (IDA) mode. The source parameters are set as follows: ion source gas 1 (GAS1), 50 psi; Ion source gas 2 (GAS2), 50 psi; Curtains (CUR), 25 pounds per square inch; Temperature (transmission electron microscope) 550 ° C; De clustering potential (DP) of 60 V or -60 V in positive or negative mode, respectively; And ion spray voltage floating (ISVF), which is 5000 V or -4000 V in positive or negative mode respectively. The TOF MS scanning parameters are set as follows: mass range, 50-1000 Da; Accumulated time 200 ms; The product ion scanning parameters are set as follows: mass range 25-1000 Da; Accumulated time 40 ms; Collision energy, with positive and negative modes of 30 or -30 V respectively; Collision energy diffusion, 15; Resolution, unit; Charging status, 1:1; Intensity, 100 CPS; Exclude isotopes within 4 Da; Quality tolerance, 50 ppm.

**Transcriptomics analysis**

Total RNA was extracted from the IPEC-J2 cells using the TRIzol reagent (Thermofisher, 15596018) following the manufacturer’s guidelines. RNA library construction, sequencing, and data analysis were performed as described in our previous study (Liu et al. 2024). Differential expression analysis of genes was performed using DESeq2 software for two distinct groups. Significantly different genes were identified with a *p*-value < 0.05. Enrichment analysis of significantly different genes was performed with Gene Ontology (GO) and KEGG analysis.

## References

Liu, Shiqi, Man Du, Yuang Tu, Wenjing You, Wentao Chen, Guoliang Liu, Junyue Li, *et al*. 2023. “Fermented mixed feed alters growth performance, carcass traits, meat quality and muscle fatty acid and amino acid profiles in finishing pigs.” *Animal Nutrition* 12: 87−95. <https://doi.org/10.1016/j.aninu.2022.09.003>

Liu, Shiq, Yuang Tu, Jiabao Sun, Peiran Cai, Yanbing Zhou, Yuqin Huang, Shu Zhang, *et al*. 2023b. “Fermented mixed feed regulates intestinal microbial community and metabolism and alters pork flavor and umami.” *Meat Science* 201: 109177. <https://doi.org/https://doi.org/10.1016/j.meatsci.2023.109177>.

Liu, Shiqi, Shiyuan Hua, Xin Gu, Peiran Cai, Yanbing Zhou, Yizhen Wang, Min Zhou, Tizhong Shan. 2024. “Production of sodium alginate-gelatin composite hydrogel-based 3D cultured fat with low cholesterol and high polyunsaturated fatty acids.” *Food Hydrocolloids* 154: 110156. https://doi.org/https://doi.org/10.1016/j.foodhyd.2024.110156

## Supplementary figures





**Figure S1 *B. subtilis* alleviates intestinal inflammation and improves antioxidant capacity.** (A) Initial body weights (*n* = 5). (B) Final body weights (*n* = 5). (C) Spleen weights (*n* = 5). (D) The statistical analysis results of the protein expression in jejunal tissues (*n* = 3). (E) The statistical analysis results of the protein expression in the colon (*n* = 3). (F) Relative mRNA expression of *Tnfa* and *Il-6* in colon tissues (*n* = 5). (G) Representative jejunal histology images by H&E staining. (H) Serum immunoglobulin levels (*n* = 6). (I) Antioxidant indicators in the colon (*n* = 6). Statistical analysis was performed using a one‐way analysis of variance (ANOVA) followed by Fisher's least significant difference test. The data are shown as means ± SEMs. BS, *B. subtilis*; *Il-6*, interleukin-6; H&E, hematoxylin and eosin; LPS, lipopolysaccharide; *Tnfα*, tumor necrosis factor α.

**

**

**Figure S2 *B. subtilis* treatment alters gut microbial composition and increases the abundance of beneficial bacteria.** (A) Principal coordinate analysis (PCoA). (B) The composition of gut microbiota at the phylum level. (C) The composition of gut microbiota at the genus level. (D) Phylogenetic tree based on the top 100 genera. (E) Heatmap of the relative abundance at the genus level. (F) PICRUSt analyses between the LPS and LPS + BS groups. (G) The correlation analysis between significantly differential genera and the genes expression level (*n* = 5). PICRUSt, Phylogenetic Investigation of Communities by Reconstruction of Unobserved States.





**Figure S3 Enhancement of intestinal epithelial barrier function by *B. subtilis* was primarily associated with metabolites.** (A) Relative mRNA levels of *Tnfα* and *Il-6* (*n* = 5). (B) Effects of supernatant derived from *B. subtilis* on the relative mRNA expression (*n* = 5). (C-D) Effects of HI BS on the protein levels of Occludin and ZO-1 (*n* = 3). Statistical analysis was performed using a one‐way analysis of variance (ANOVA) followed by Fisher's least significant difference test. The data are shown as means ± SEMs. HI BS, heat-inactivated *B. subtilis.*





**Figure S4 Analysis of metabolites derived from *B. subtilis.*** (A) Z-score plot of the top 50 different metabolites with the highest VIP values. (B) Correlation network diagram of the top 50 different metabolites with the highest VIP values. The pink lines represent positive correlations, while the blue lines indicate negative correlations. The thickness of the lines corresponds to the magnitude of the absolute correlation coefficients, with thicker lines signifying stronger correlations. (C) Relative abundance of SCFAs. Statistical analysis was performed using an independent samples t-test. The data are shown as means ± SEMs (*n* = 3). SCFAs, short-chain fatty acids.





**Figure S5 Effects of metabolites derived from *B. subtilis* on intestinal tight junction proteins.** (A) Experimental design from metabolites derived from *B. subtilis* co-cultured with IPEC-J2 cells. (B-C) Protein levels of Occludin and ZO-1 (*n* = 3). (D-E) Effects of HMP on the protein levels of Occludin in jejunum (*n* = 3). Statistical analysis was performed using a one‐way analysis of variance (ANOVA) followed by Fisher's least significant difference test. The data are shown as means ± SEMs. HH, high-dose HMP; LH, low-dose HMP.

**

**

**Figure S6 Gadd45A is a key regulator of intestinal barrier integrity.** (A) Heatmap of differentially expressed genes in DSS colitis mouse models (GSE168053). Correlation analyses of *Gadd45a* and *ZO-1* and *Occludin* expression patterns. (B) Heatmap of gene expression in colon samples from the healthy and UC patients (GSE224758). (C) Heatmap of gene expression in Ochratoxin A-induced Caco-2 cell injury models (GSE115081). (D) Relative mRNA levels of *Gadd45a* in jejunal tissues (*n* = 5). (E-F) Effects of *B. subtilis* on the protein levels of GADD45A in jejunum (*n* = 3). (G-H) Effects of HMP on the protein levels of GADD45A in the jejunum (*n* = 3). Statistical analysis was performed using a one‐way analysis of variance (ANOVA) followed by Fisher's least significant difference test. The data are shown as means ± SEMs. DSS, dextran sulfate sodium; UC, ulcerative colitis.

**

**

**Figure S7 Effects of GADD45A deficiency on intestinal epithelial barrier.** (A) Representative histology images by H&E staining in duodenum. (B) Immunohistochemical staining of pS6. (C-D) Protein levels of ZO-1, Occludin, and Claudin1 (*n* = 3). Statistical analysis was performed using a one‐way analysis of variance (ANOVA) followed by Fisher's least significant difference test. The data are shown as means ± SEMs. shG45A, knockdown of *Gadd45a*; pS6, S6 ribosomal protein phosphorylation.





**Figure S8 GADD45A enhanced intestinal epithelial barrier dependent on Wnt/β-catenin signaling pathway.** (A) A heatmap of mRNA expression profiles (*n* = 3). (B) A volcano plot showing the differential genes. (C) Bubble plots showing the top enriched 30 items based on differentially upregulated genes. (D) Bubble plots showing the top enriched 30 items based on differentially downregulated genes. (E) Relative expression heatmap of genes in the Wnt signaling pathway. (F) Relative expression heatmap of genes in the mTOR signaling pathway. (G) Relative mRNA levels of *Gadd45a* and genes related to Wnt signaling pathway between Overexpression of *Gadd45a* (oe-G45A) and control group (*n* = 3). (H) Western blot analysis of Wnt and mTOR signaling pathway. (I) After supplementing control and sh-GADD45A *adenovirus* for 36 h, cells were treated with a Wnt agonist BML-284 for 24 h. (J) Relative mRNA levels of *Occludin*, *ZO-1*, *ZO-2*, and *Claudin1* (*n* = 4). (K) Western blot analysis of ZO-1 in the presence of BML-284. (L) Western blot analysis of ZO-1 in the presence of an inhibitor LF3. Statistical analysis was performed using an independent samples *t*-test. The data are shown as means ± SEMs. Ctrl, Control; shG45A, knockdown of *Gadd45a*; oe-G45A, overexpression of *Gadd45a*.
